# A systematic review of the use of theory in the design of guideline dissemination and implementation strategies and interpretation of the results of rigorous evaluations

**DOI:** 10.1186/1748-5908-5-14

**Published:** 2010-02-09

**Authors:** Philippa Davies, Anne E Walker, Jeremy M Grimshaw

**Affiliations:** 1 Health Services Research Unit, University of Aberdeen, UK; 2 Clinical Epidemiology Program, Ottawa Health Research Institute and Department of Medicine, University of Ottawa, 1053 Carling Avenue, Administration Building, Room 2-017, Ottawa ON K1Y 4E9, Canada

## Abstract

**Background:**

There is growing interest in the use of cognitive, behavioural, and organisational theories in implementation research. However, the extent of use of theory in implementation research is uncertain.

**Methods:**

We conducted a systematic review of use of theory in 235 rigorous evaluations of guideline dissemination and implementation studies published between 1966 and 1998. Use of theory was classified according to type of use (explicitly theory based, some conceptual basis, and theoretical construct used) and stage of use (choice/design of intervention, process/mediators/moderators, and post hoc/explanation).

**Results:**

Fifty-three of 235 studies (22.5%) were judged to have employed theories, including 14 studies that explicitly used theory. The majority of studies (n = 42) used only one theory; the maximum number of theories employed by any study was three. Twenty-five different theories were used. A small number of theories accounted for the majority of theory use including PRECEDE (Predisposing, Reinforcing, and Enabling Constructs in Educational Diagnosis and Evaluation), diffusion of innovations, information overload and social marketing (academic detailing).

**Conclusions:**

There was poor justification of choice of intervention and use of theory in implementation research in the identified studies until at least 1998. Future research should explicitly identify the justification for the interventions. Greater use of explicit theory to understand barriers, design interventions, and explore mediating pathways and moderators is needed to advance the science of implementation research.

## Background

There is growing interest in the use of cognitive, behavioural, and organisational theories to understand barriers to implementation, to inform the design of interventions to improve professional practice, and to explore the mediating mechanisms and potential moderators of such interventions in the context of rigorous evaluations [1,2]. However, despite this interest, the extent to which theory has been used in implementation research is unclear. To address this issue, we conducted a systematic review of the use of theory linked to a large systematic review of the effects of guideline dissemination and implementation strategies [3]. Specifically, we were interested in the extent that theory was used in the design of guideline dissemination and implementation interventions, and interpretation of their controlled evaluations.

## Methods

We examined the use of theory in studies identified in a systematic review of rigorous evaluations of clinical practice guideline dissemination and implementation strategies. The full methods and results of the systematic review are available elsewhere [3]. Briefly, we searched Medline, EMBASE, Health Star, the Cochrane Controlled Trials Register, and SIGLE (System for Information on Grey Literature in Europe) using a highly sensitive search strategy developed for the Cochrane Effective Practice and Organisation of Care (EPOC) group between 1976 and 1998 [4]. Searches were not restricted by language or publication type. We included cluster and individual randomized controlled trials, controlled clinical trials, controlled before and after studies, and interrupted time series that evaluated any guideline dissemination or implementation strategy targeting physicians and that reported an objective measure of provider behavior and/or patient outcome. Two reviewers independently screened the search results and assessed studies against the inclusion criteria. Disagreements were resolved by consensus. The final sample included 285 reports of 235 studies yielding 309 comparisons of guideline dissemination and implementation strategies.

For the purposes of the current study, we identified whether included studies had used a theory to inform the design of an intervention and/or the interpretation of the results. A study was considered to have used a theory if the authors stated that they had done so within the report of the study, preferably with a source reference and/or an explanation of how the theory was proposed to explain the phenomenon to which it had been applied. Where a study described a framework or approach that appeared to be theoretically based, but the authors had not explicitly stated that they had used a theory, a decision was made by two reviewers regarding whether or not the study should be classified as theory-based or not. PD read all papers to identify whether or not they used theory. In cases of uncertainty, papers were considered by a second reviewer (AW), and a consensus was reached about whether or not these studies should be classified as having used theory or not.

We classified all papers using a descriptive framework that considered the level of theory use and the stage at which theory was used (Appendix 1). Level of theory use reflects the intensiveness of use of theory within studies. Studies judged to have used theories were classified within the first two categories ('explicitly theory based'; 'some conceptual basis'). Studies using individual constructs from theories, *e.g*., knowledge, attitude, self-efficacy, that were not reported within a theoretical framework were classified as 'construct(s) (unrelated to theory)'. In cases where a study employed more than one theory, each instance of theory use was classified separately using the framework. Studies in the first three categories--explicitly theory-based; some conceptual basis; constructs (unrelated to theory)--were further classified according to the stage of the research process at which the theory (or construct) was used. For the purposes of the review, the stage of use categories were treated as being mutually exclusive, *i.e*., each instance of theory use was assigned to one category only (the first stage of the research process at which the theory has been integrated). This is not to say that studies employing a theory at one stage could not, or did not, use the same theory at any other stage.

## Results

Fifty-three of 235 studies (22.5%) were judged to have employed theories of behaviour or behaviour change (see Additional File 1) [5-67]. Of these, fourteen did so explicitly and thirty-nine were considered to have some conceptual basis. A further ten studies used individual constructs from theories only. The majority of studies (n = 42) used only one theory. The maximum number of theories employed by any study was three. The remaining 172 studies were judged to have not employed theories of behaviour or constructs and were not studied further. Brief descriptions of the identified theories are provided in Additional File 2.

Twenty-five different theories representing 66 occasions of theory use were found (Table 1). A small number of theories accounted for the majority of theory use. For example, PRECEDE (Predisposing, Reinforcing, and Enabling Constructs in Educational Diagnosis and Evaluation) [68], diffusion of innovation [69], information overload [70], and social marketing (academic detailing) [71] accounted for just over half of all instances of theory use. Fourteen studies used 11 theories explicitly. Only two theories were used explicitly more than once. The PRECEDE theory was also the most commonly employed theory within the review as a whole across all levels of theory use. Thirty-nine studies used sixteen theories within some conceptual basis. For two of the most commonly employed theories (diffusion of innovation, social marketing (academic detailing)) all instances of their use were in this category. Theory was used during the intervention choice/design stage 49 times (74.2%), for process/mediator/moderator analyses seven times (10.6%) and for post hoc explanation 10 times (16.6%).

**Table 1:** Level of use of theory within studies (including level of theory use)

Theory	Used explicitly	Used with some conceptual basis	Total
PRECEDE	3	8	11
Diffusion of innovation	0	8	8
Information overload	1	7	8
Academic detailing	0	8	8
Social cognitive theory	0	4	4
Theory of reasoned action	1	2	3
Social influence	0	2	2
Social learning theory	0	2	2
Behaviour modification techniques	1	1	2
Continuous quality improvement	2	0	2
Field theory	0	2	2
Cybernetic theory	1	0	1
Dual task theory	0	1	1
Elaboration likelihood model	1	0	1
Four-step intervention	0	1	1
Goals, Emotions and personal capabilities theory	0	1	1
Health belief model	0	1	1
Learning styles	0	1	1
Organizational development	1	0	1
Patient care appraisal model	1	0	1
Rule-based expert system	0	1	1
Shot-gun method	0	1	1
Stages of change	0	1	1
Treatment theory	1	0	1
Vividness criterion (human inference theory)	1	0	1

**Total**	**14**	**52**	**66**

Twenty-four studies used individual constructs from theory (Additional File 1) including knowledge (17 studies), attitude (14 studies), and self-efficacy (two studies). All studies used constructs in process/mediator/moderator analyses (although few of the studies carried out formal tests for the mediating or moderating effects of these variables). The rationale for why specific theories and constructs were used was not apparent in the majority of studies.

## Discussion

This study examined the use of theories within a large sample of rigorous evaluations of guideline dissemination and implementation strategies published before 1998. We observed that the minority of studies (22.5%) reported any use of theory, although less than 6% explicitly used theory. Theory was most often used to inform the choice and design of interventions (although this may be in part due to our approach to mutually exclusive coding of the stage of theory use). Theoretical constructs were used in specific process/mediator/moderator analyses. There was poor reporting of the rationale for using specific theories and constructs. In the case of many of the theory-based studies considered in this review, it was difficult to determine the quality of theory use (*i.e*., the extent to which researchers had employed the theory with fidelity), although this was not one of the objectives of our review.

To our knowledge, this is the first review of the use of theory in implementation intervention studies. The use of studies identified for a rigorous systematic review of guideline dissemination and implementation strategies ensures a comprehensive and representative sample. However, we did not explicitly look for published process evaluations alongside the identified studies that might be more likely to report theoretical considerations. A further meta-synthesis of qualitative studies of general practitioners' experiences and attitudes towards the use of clinical practice guidelines only found 12 studies all published between 1998 and 2006 [72]. The focus of the original review on practice guideline and dissemination studies targeting medically qualified healthcare professionals ensures that we cannot comment on whether the use of theory was greater in dissemination and implementation studies focussing on studies of behaviour change interventions other then practice guidelines or targeting other stakeholders. Further, the timeframe of the searches for the systematic review means that we cannot comment on whether use of theory has increased in studies published since 1998. Although it is only in the last five years that there has been greater discourse about the role of theory in implementation research [2,73]. We would encourage researchers to treat this as baseline data and replicate this review for studies published since 1998 to explore whether there have been any improvements in the use of theory in implementation research.

PD undertook the first screen of studies to identify whether or not they used theory. This could have introduced some misclassification of studies. We tried to reduce this risk by having a low threshold for consulting AW if PD was in anyway uncertain. It would clearly have been better if two reviewers had independently screened all studies. However, this was not possible given the resources available to us.

It is possible that authors may have used theory in more of the studies, but not reported it in the main study publications due to space constraints or lack of recognition of importance of explicitly outlining the rationale for interventions [1]. The increased emphasis on greater transparency in scientific reporting (for example, publishing study protocols) and the availability of online journals (such as Implementation Science) without space constraints could address this issue.

It was challenging to identify and classify theories given the paucity of description. Some readers may argue whether some of our categories of theory are actual theories. Nevertheless, removing some categories would further reduce the number of studies that provided an explicit rationale for their interventions.

## Conclusion

Greater use of explicit theory to understand barriers, design interventions, and explore mediating pathways and moderators has been advocated to advance of the science of implementation research [1]. This study highlights the lack of use of theory until at least 1998.

It is recommended that researchers conducting theory-based studies give careful consideration to the choice of theory used and develop a clear rationale of how the theory is proposed to operate within the study. Where possible, hypotheses deduced from the theory to design the study should be explicitly examined. Reports of theory-based research should be explicit about all theories used including, where appropriate, citations to original literature relating to the theories. The way in which the theory is proposed to explain that to which it has been applied should be clearly stated, as should methodological detail relating to the way in which the theory has been operationalised and analysed.

## Competing interests

The authors declare that they have no competing interests.

## Authors' contributions

PD, AW, JG conceived the study. PD and AW abstracted data from paper. PD wrote first draft of paper. AW and JG commented on drafts of paper. All authors read and approved the final manuscript.

## Appendix 1. Descriptive framework used to classify studies

### Level of use

#### Explicitly theory-based

Study explicitly stated a theory and provided a direct test of one or more of the hypotheses deduced from a named theory in order to design the study. Hence, it was possible to examine the suitability of the explanation provided by the theory for the intervention to which it had been applied.

#### Some conceptual basis

Studies classified as having some conceptual basis were those where a theory was judged to have been used within the study, but where the study did not provide a test of any of the hypotheses deduced from the theory in order to design the study. Studies included in this category were those where the authors stated that they had employed a theory within the study, or where the study described a framework or approach that appeared to be theoretically-based and two reviewers (PD, AW) agreed that the study should be considered to be theory-based.

#### Theoretical construct used

Studies included in this category are those where one or more constructs were examined within the study, but the use of constructs was not embedded within the framework of a theory. Where a construct was referred to within the context of a theory, but was the only component of the theory that was measured and considered, this was considered to be use of the theory within the 'some conceptual basis' category.

### Stage of use

#### Choice/design of intervention

The choice/design category refers to the use of theory to guide the choice of intervention, such as, for example, to understand the reasons for the observed gap between clinical practice and the guideline recommendations, or the use of theory to guide the design of the intervention used to implement the guidelines.

#### Process/mediators/moderators

This category refers to the use of theories or constructs for the purposes of process assessment, or to explore mediators or moderators of behaviour or the effects of the intervention. Studies classified as using constructs (unrelated to theory) were further classified as 'process,' 'mediator,' or 'moderator'. These further classifications were based on the following descriptions:

##### Process

Where a construct has been measured once or more (*e.g*., pre- and post-intervention, or post-intervention in a study group and a control group), but has not been analysed in relation to any other variables measured within the study.

##### Moderator

Where a construct has been measured once or more and analysed in relation to outcome variables

##### Mediator

In order to demonstrate the mediating effect of the construct it should be measured pre- and post-intervention (or post-intervention only in both a control and study group) and changes in the construct should be analysed in relation to changes in outcome measures obtained within the study.

#### Post hoc/explanation

This category refers to retrospective use of theory to explain the results of the study or to stimulate further discussion. Whilst the use of theories within this category might appear to overlap with the previous categories (*i.e*., a theory might be employed to reflect on the design of the intervention or potential mediators or moderators of its effectiveness), the distinguishing feature of this category is that the theory has been introduced after the intervention has been carried out.

## Supplementary Material

Additional file 1 **Use of theories and constructs in studies**. Details of the studies that used theories (and constructs), the theories and constructs used and level and stage of use. Click here for file

Additional file 2 **Glossary of theories/frameworks used**. Brief descriptions of the identified theories and frameworks. Click here for file
