# Supplementary material for: A systematic review of the use of theory in the design of guideline dissemination and implementation strategies and interpretation of the results of rigorous evaluations
Source: Implement Sci. 2010 Feb 9;5:14. doi: 10.1186/1748-5908-5-14 (PMC2832624; doi:10.1186/1748-5908-5-14)
Supplement: Additional file 1 — Use of theories and constructs in studies. Details of the studies that used theories (and constructs), the theories and constructs used and level and stage of use. [file 1748-5908-5-14-S1.DOC]

## Additional File 1. Use of theories and constructs in studies

| **Study** | **Theory/Construct** | **Level of Use** | **Stage of Use** |
| --- | --- | --- | --- |
| Anonymous 1996 [1] | Attitude | Construct | Process/mediator/moderator |
| Avorn 1992 [2] | Social Marketing (Academic Detailing) | Some conceptual basis | Choice/design |
| Banks 1988 [3] | Information Overload | Some conceptual basis | Choice/design |
| Boissel 1995 [4] | Knowledge | Construct | Process/mediator/moderator |
| Caggiula 1996 [5] | Social Cognitive Theory  (Self-efficacy) | Some conceptual basis | Choice/design |
| Calkins1995 [6] | Diffusion of Innovation  Field Theory  Knowledge  Attitude | Some conceptual basis  Some conceptual basis  Construct  Construct | Choice/design  Choice/design  Process/mediator/moderator  Process/mediator/moderator |
| Callahan 1994 [7] | PRECEDE | Some conceptual basis | Choice/design |
| Carney 1992 [8] | PRECEDE  Social Cognitive Theory  Knowledge | Some conceptual basis  Some conceptual basis  Construct | Choice/design  Choice/design  Process/mediator/moderator |
| Cohen 1985 [9] | Theory of Reasoned Action  Knowledge | Some conceptual basis  Construct | Process/mediator/moderator  Process/mediator/moderator |
| Constanza 1992 [10] | PRECEDE | Explicitly theory based | Process/mediator/moderator |
| De Burgh 1995 [11] | Social Marketing (Academic Detailing) | Some conceptual basis | Choice/design |
| Dempsey 1995 [12] | Continuous Quality Improvement | Explicitly theory based | Choice/design |
| Diwan 1995 [13] | Social Marketing (Academic Detailing)  Attitude | Some conceptual basis  Construct | Choice/design  Process/mediator/moderator |
| Elliot 1997 [14] | Social Learning Theory  Diffusion of Innovation  Theory of Reasoned Action  Knowledge | Some conceptual basis  Some conceptual basis  Some conceptual basis  Construct | Choice/design  Choice/design  Process/mediator/moderator  Process/mediator/moderator |
| Evans 1996 [15] | Social Cognitive Theory  (self-efficacy)  Knowledge  Attitude | Some conceptual basis  Construct  Knowledge | Choice/design  Process/mediator/moderator  Process/mediator/moderator |
| Evans 1997 [16] | Field Theory | Some conceptual basis | Choice/design |
| Feder 1995 [17] | Social Marketing (Academic Detailing) | Some conceptual basis | Choice/design |
| Fender 1999 [18] | Social Marketing (Academic Detailing) | Some conceptual basis | Choice/design |
| Fletcher 1993 [19] | PRECEDE | Explicitly theory based | Choice/design |
| Flynn 1997 [20] | PRECEDE | Some conceptual basis | Choice/design |
| Gemson 1995 [21] | Health Belief Model  Knowledge | Some conceptual basis  Construct | Post-hoc explanation/discussion  Process/mediator/moderator |
| Goldberg 1998 [22] | Continuous Quality Improvement  Social Marketing (Academic Detailing) | Explicitly theory based  Some conceptual basis | Choice/design  Choice/design |
| Gortmaker 1998 [23] | Social Learning Theory | Some conceptual basis | Choice/design |
| Gorton 1995 [24] | Diffusion of Innovation  Social Influence | Some conceptual basis  Some conceptual basis | Post-hoc explanation/discussion  Post-hoc explanation/discussion |
| Grady 1997 [25] | Behaviour modification | Explicitly theory based | Choice/design |
| Headrick 1992 [26] | Knowledge  Attitudes | Construct  Construct | Process/mediator/moderator  Process/mediator/moderator |
| Herman 1994 [27] | Knowledge  Self efficacy | Construct  Construct | Process/mediator/moderator  Process/mediator/moderator |
| Hobbs 1966 [28] | Rule-based expert system  Knowledge | Some conceptual basis  Construct | Choice/design  Process/mediator/moderator |
| Hulscher 1997 [29] | Social Marketing (Academic Detailing)  4-step Intervention | Some conceptual basis  Some conceptual basis | Choice/design  Choice/design |
| Keyserling 1997 [30] | Social Cognitive Theory  (self-efficacy) | Some conceptual basis | Choice/design |
| Leviton 1999 [31] | Social Influence  Diffusion of Innovation | Some conceptual basis  Some conceptual basis | Choice/design  Post-hoc discussion/explanation |
| Lin 1997 [32] | PRECEDE  Diffusion of Innovation | Some conceptual basis  Some conceptual basis | Choice/design  Post-hoc discussion/explanation |
| Litzelman 1993 [33] | Dual Task Theory | Some conceptual basis | Choice/design |
| Lobach 1997 [34] | Information Overload | Some conceptual basis | Choice/ design |
| Lomas 1989 [35] | Diffusion of Innovation  Attitude  Knowledge | Some conceptual basis  Construct  Construct | Choice/design  Process/mediator/moderator  Process/mediator/moderator |
| Marton 1995 [36] | Knowledge  Attitudes | Construct  Construct | Process/mediator/moderator  Process/mediator/moderator |
| Mazzuca 1988 [37] | Behaviour modification | Some conceptual basis | Choice/design |
| McDonald 1976 [38] | Information Overload | Some conceptual basis | Choice/design |
| McDonald 1980 [39] | Information Overload | Explicitly theory based | Choice/design |
| McDonald 1984 [40] | Information Overload  Attitude | Some conceptual basis  Construct | Choice/design  Process/mediator/moderator |
| Nalven 1997 [41] | Knowledge | Construct | Process/mediator/moderator |
| Nattinger 1989 [42] | Knowledge | Construct | Process/mediator/moderator |
| Ockene 1994 [43] | Knowledge  Self efficacy | Construct  Construct | Process/mediator/moderato  Process/mediator/moderator |
| Onion 1997[44] | Elaboration Likelihood Model  Learning Styles  Goals, emotions, and personal capability beliefs  Attitude  Knowledge | Explicitly theory based  Some conceptual basis  Some conceptual basis  Construct  Construct | Choice/design  Process/mediator/moderator  Choice/design  Process/mediator/moderator  Process/mediator/moderator |
| Overhage 1997 [45] | Information Overload | Some conceptual basis | Choice/design |
| Palmer 1985 [46] | Theory-driven evaluation | Explicitly theory based | Process/mediator/moderator |
| Putnam 1985 [47] | Patient Care Appraisal Model | Explicitly theory based | Choice/design |
| Raisch 1990 [48] | Human Inference (Vividness Criterion) | Explicitly theory based | Choice/design |
| Restuccia 1982 [49] | Cybernetic theory | Explicitly theory based | Choice/design |
| Rogers 1982 [50] | Information Overload | Some conceptual basis | Choice/design |
| Sommers 1984 [51] | Organizational development  Information overload | Explicitly theory based  Some conceptual basis | Choice/design  Choice/design |
| Soumerai 1993 [52] | Social Marketing (Academic Detailing) | Some conceptual basis | Choice/design |
| Soumerai 1998 [53] | Diffusion of Innovation  PRECEDE | Some conceptual basis  Some conceptual basis | Choice/design  Post-hoc explanation/discussion |
| Steffensen 1997 [54] | Diffusion of Innovation  Attitude  Knowledge | Some conceptual basis  Construct  Construct | Post-hoc explanation/discussion  Process/mediator/moderator  Process/mediator/moderator |
| Studnicki 1997 [55] | PRECEDE | Some conceptual basis | Post-hoc explanation/discussion |
| Thamer 1998 [56] | PRECEDE | Some conceptual basis | Post-hoc explanation/discussion |
| Turner 1994 [57] | Stages of Change  Attitude | Some conceptual basis  Construct | Post-hoc explanation/discussion  Process/mediator/moderator |
| Urban 1995 [58] | PRECEDE | Explicitly theory based | Choice/design |
| Van Der Weijden 1999 [59] | PRECEDE | Some conceptual basis | Process/mediator/moderator |
| Van Essen 1997 [60] | Shot-gun method | Some conceptual basis | Choice/design |
| Vinicor 1987 [61] | Theory of Reasoned Action  Knowledge | Explicitly theory based  Construct | Process/mediator/moderator  Process/mediator/moderator |
| Vissers 1995 [62] | Attitude | Construct | Process/mediator/moderator |
| Watson 1998 [63] | Attitude | Construct | Process/mediator/moderator |

**References for Additional File 1**

1. Anonymous. CCQE-AHCPR guideline criteria project. Building and applying a guideline-based performance measurement system: develop, apply, and evaluate medical review criteria and educational outreach based upon practice guidelines : final project report. 97-N002 - 97-N003, U-97. 1996. Rockville, MD, U.S. Dept. of Health and Human Services, Public Health Service, Agency for Health Care Policy and Research.

2. Avorn J, Soumerai S, Everitt D, Ross-Degnan D, Beers M, Sherman D *et al*.: **A randomized trial of a program to reduce the use of psychoactive drugs in nursing homes.** *New England Journal of Medicine* 1992, **327:** 168-173.

3. Banks N, Palmer R, Kane N, Braun O, Feldstein M, Harrington A: **Implementation and evaluation of a computerized reminder system in ambulatory care.** *Proc Ann Symp Comput Applic Med Care* 1988, **12:** 753-757.

4. Boissel J, Collet J, Alborini A, Cordel J, Filsnoel J, Gillet J *et al*.: **Education program for general practitioners on breast and cervical cancer screening: a randomized trial. PRE.SA.GF Collaborative Group.** *Revue d Epidemiologie et de Sante Publique* 1995, **43:** 541-547.

5. Caggiula A, Watson J, Kuller L, Olson M, Milas N, Berry M *et al*.: **Cholesterol-lowering intervention program. Effect of the step I diet in community office practices.** *Archives of Internal Medicine* 1996, **156:** 1205-1213.

6. Calkins E, Katz L, Karuza J, Wagner A: **The small group consensus process for changing physician practices: influenza vaccination.** *HMO Practice* 1995, **9:** 107-110.

7. Callahan C, Hendrie H, Dittus R, Brater D, Hui S, Tierney W: **Improving treatment of late life depression in primary care: a randomized clinical trial.** *Journal of the American Geriatrics Society* 1994, **42:** 839-846.

8. Carney P, Dietrich A, Keller A, Landgraf J, O'Connor G: **Tools, teamwork, and tenacity: an office system for cancer prevention.** *Journal of Family Practice* 1992, **35:** 388-394.

9. Cohen S, Weinberger M, Hui S, Tierney W, McDonald C: **The impact of reading on physicians' nonadherence to recommended standards of medical care.** *Social Science & Medicine* 1985, **21:** 909-914.

10. Costanza M, Zapka J, Harris D, Hosmer D, Barth R, Gaw V *et al*.: **Impact of a physician intervention program to increase breast cancer screening.** *Cancer Epidemiology, Biomarkers & Prevention* 1992, **1:** 581-589.

11. de Burgh S, Mant A, Mattick R, Donnelly N, Hall W, Bridges-Webb C: **A controlled trial of educational visiting to improve benzodiazepine prescribing in general practice.** *Australian Journal of Public Health* 1995, **19:** 142-148.

12. Dempsey C: **Nursing home-acquired pneumonia: outcomes from a clinical process improvement program.** *Pharmacotherapy* 1995, **15:** 33S-38S.

13. Diwan V, Wahlstrom R, Tomson G, Beermann B, Sterky G, Eriksson B: **Effects of "group detailing" on the prescribing of lipid-lowering drugs: a randomized controlled trial in Swedish primary care.** *Journal of Clinical Epidemiology* 1995, **48:** 705-711.

14. Elliott T, Murray D, Oken M, Johnson K, Braun B, Elliott B *et al*.: **Improving cancer pain management in communities: main results from a randomized controlled trial.** *Journal of Pain & Symptom Management* 1997, **13:** 191-203.

15. Evans A, Rogers L, Peden JG J, Seelig C, Layne R, Levine M *et al*.: **Teaching dietary counseling skills to residents: patient and physician outcomes. The CADRE Study Group.** *American Journal of Preventive Medicine* 1996, **12:** 259-265.

16. Evans D, Mellins R, Lobach K, Ramos-Bonoan C, Pinkett-Heller M, Wiesemann S *et al*.: **Improving care for minority children with asthma: professional education in public health clinics.** *Pediatrics* 1997, **99:** 157-164.

17. Feder G, Griffiths C, Highton C, Eldridge S, Spence M, Southgate L: **Do clinical guidelines introduced with practice based education improve care of asthmatic and diabetic patients? A randomised controlled trial in general practices in east London.** *BMJ* 1995, **311:** 1473-1478.

18. Fender G, Prentice A, Gorst T, Nixon R, Duffy S, Day N *et al*.: **Randomised controlled trial of educational package on management of menorrhagia in primary care: the Anglia menorrhagia education study.** *BMJ* 1999, **318:** 1246-1250.

19. Fletcher S, Harris R, Gonzalez J, Degnan D, Lannin D, Strecher V *et al*.: **Increasing mammography utilization: a controlled study.** *Journal of the National Cancer Institute* 1993, **85:** 112-120.

20. Flynn B, Gavin P, Worden J, Ashikaga T, Gautam S, Carpenter J: **Community education programs to promote mammography participation in rural New York State.** *Preventive Medicine* 1997, **26:** 102-108.

21. Gemson D, Ashford A, Dickey L, Raymore S, Roberts J, Ehrlich M *et al*.: **Putting prevention into practice. Impact of a multifaceted physician education program on preventive services in the inner city.** *Archives of Internal Medicine* 1995, **155:** 2210-2216.

22. Goldberg H, Wagner E, Fihn S, Martin D, Horowitz C, Christensen D *et al*.: **Putting prevention into practice. Impact of a multifaceted physician education program on preventive services in the inner city.** *Joint Commission Journal on Quality Improvement* 1998, **24:** 130-142.

23. Gortmaker S, Bickford A, Mathewson H, Dumbaugh K, Tirrell P: **A successful experiment to reduce unnecessary laboratory use in a community hospital.** *Medical Care* 1988, **26:** 631-642.

24. Gorton T, Cranford C, Golden W, Walls R, Pawelak J: **Primary care physicians' response to dissemination of practice guidelines.** *Archives of Family Medicine* 1995, **4:** 135-142.

25. Grady K, Lemkau J, Lee N, Caddell C: **Enhancing mammography referral in primary care.** *Preventive Medicine* 1997, **26:** 791-800.

26. Headrick L, Speroff T, Pelecanos H, Cebul R: **Efforts to improve compliance with the National Cholesterol Education Program guidelines. Results of a randomized controlled trial.** *Archives of Internal Medicine* 1992, **152:** 2490-2496.

27. Herman C, Speroff T, Cebul R: **Improving compliance with immunization in the older adult: results of a randomized cohort study.** *Journal of the American Geriatrics Society* 1994, **42:** 1154-1159.

28. Hobbs F, Delaney B, Carson A, Kenkre J: **A prospective controlled trial of computerized decision support for lipid management in primary care.** *Fam Pract* 1996, **13:** 133-137.

29. Hulscher M, van Drenth B, van der Wouden J, Mokkink H, van Weel C, Grol R: **Changing preventive practice: a controlled trial on the effects of outreach visits to organise prevention of cardiovascular disease.** *Quality in Health Care* 1997, **6:** 19-24.

30. Keyserling T, Ammerman A, Davis C, Mok M, Garrett J, Simpson R Jr: **A randomized controlled trial of a physician-directed treatment program for low-income patients with high blood cholesterol: the Southeast Cholesterol Project.** *Archives of Family Medicine* 1997, **6:** 135-145.

31. Leviton L, Goldenberg R, Baker C, Schwartz R, Freda M, Fish L *et al*.: **Methods to encourage the use of antenatal corticosteroid therapy for fetal maturation: a randomized controlled trial.** *JAMA* 1999, **281:** 46-52.

32. Lin E, Katon W, Simon G, Von Korff M, Bush T, Rutter C *et al*.: **Achieving guidelines for the treatment of depression in primary care: is physician education enough?** *Medical Care* 1997, **35:** 831-842.

33. Litzelman D, Slemenda C, Langefeld C, Hays L, Welch M, Bild D *et al*.: **Reduction of lower extremity clinical abnormalities in patients with non-insulin-dependent diabetes mellitus. A randomized, controlled trial.** *Annals of Internal Medicine* 1993, **119:** 36-41.

34. Lobach D, Hammond W: **Computerized decision support based on a clinical practice guideline improves compliance with care standards.** *American Journal of Medicine* 1997, **102:** 89-98.

35. Lomas J, Anderson GM, Domnick-Pierre K, Vayda E, Enkin MW, Hannah WJ: **Do practice guidelines guide practice? The effect of a consensus statement on the practice of physicians.** *N Engl J Med* 1989, **321:** 1306-1311.

36. Marton K, Tul V, Sox HC J: **Modifying test-ordering behavior in the outpatient medical clinic. A controlled trial of two educational interventions.** *Archives of Internal Medicine* 1985, **145:** 816-821.

37. Mazzuca S, Vinicor F, Cohen S, Norton J, Fineberg N, Fineberg S *et al*.: **The Diabetes Education Study: a controlled trial of the effects of intensive instruction of internal medicine residents on the management of diabetes mellitus.** *Journal of General Internal Medicine* 1988, **3:** 1-8.

38. McDonald CJ: **Protocol-based computer reminders, the quality of care and the non-perfectability of man.** *N Engl J Med* 1976, **295:** 1351-1355.

39. McDonald CJ, Wilson GA, McCabe GP, Jr.: **Physician response to computer reminders.** *JAMA* 1980, **244:** 1579-1581.

40. McDonald C, Hui S, Smith D, Tierney W, Cohen S, Weinberger M *et al*.: **Reminders to physicians from an introspective computer medical record. A two-year randomized trial.** *Annals of Internal Medicine* 1984, **100:** 130-138.

41. Nalven L, Hofkosh D, Feldman H, Kelleher K: **Teaching pediatric residents about early intervention and special education.** *Journal of Developmental & Behavioral Pediatrics* 1997, **18:** 371-376.

42. Nattinger A, Panzer R, Janus J: **Improving the utilization of screening mammography in primary care practices.** *Archives of Internal Medicine* 1989, **149:** 2087-2092.

43. Ockene J, Adams A, Pbert L, Luippold R, Hebert J, Quirk M *et al*.: **The Physician-Delivered Smoking Intervention Project: factors that determine how much the physician intervenes with smokers.** *Journal of General Internal Medicine* 1994, **9:** 379-384.

44. Onion C: *Changes inmedical practice following superficial and deep processing of evidence: a controlled experiment in clinical guideline implementation.* University of Liverpool; 1997. PhD.

45. Overhage J, Tierney W, Zhou X, McDonald C: **A randomized trial of "corollary orders" to prevent errors of omission.** *Journal of the American Medical Informatics Association* 1997, **4:** 364-375.

46. Palmer R, Louis T, Hsu L, Peterson H, Rothrock J, Strain R *et al*.: **A randomized controlled trial of quality assurance in sixteen ambulatory care practices.** *Medical Care* 1985, **23:** 751-770.

47. Putnam R, Curry L: **Impact of patient care appraisal on physician behaviour in the office setting.** *CMAJ* 1985, **132:** 1025-1029.

48. Raisch D, Bootman J, Larson L, McGhan W: **Improving antiulcer agent prescribing in a health maintenance organization.** *American Journal of Hospital Pharmacy* 1990, **47:** 1766-1773.

49. Restuccia J: **The effect of concurrent feedback in reducing inappropriate hospital utilization.** *Medical Care* 1982, **20:** 46-62.

50. Rogers J, Haring O, Wortman P, Watson R, Goetz J: **Medical information systems: assessing impact in the areas of hypertension, obesity and renal disease.** *Medical Care* 1982, **20:** 63-74.

51. Sommers L, Sholtz R, Shepherd R, Starkweather D: **Physician involvement in quality assurance.** *Medical Care* 1984, **22:** 1115-1138.

52. Soumerai SB, Salem-Schatz S, Avorn J, Casteris CS, Ross-Degnan D, Popovsky MA: **A controlled trial of educational outreach to improve blood transfusion practice.** *JAMA* 1993, **270:** 961-966.

53. Soumerai S, McLaughlin T, Gurwitz J, Guadagnoli E, Hauptman P, Borbas C *et al*.: **Effect of local medical opinion leaders on quality of care for acute myocardial infarction: a randomized controlled trial.** *JAMA* 1998, **279:** 1358-1363.

54. Steffensen F, Sorensen H, Olesen F: **Impact of local evidence-based clinical guidelines--a Danish intervention study.** *Fam Pract* 1997, **14:** 209-215.

55. Studnicki J, Remmel R, Campbell R, Werner D: **The impact of legislatively imposed practice guidelines on cesarean section rates: the Florida experience.** *American Journal of Medical Quality* 1997, **12:** 62-68.

56. Thamer M, Ray N, Henderson S, Rinehart C, Sherman C, Ferguson J: **Influence of the NIH Consensus Conference on Helicobacter pylori on physician prescribing among a Medicaid population.** *Medical Care* 1998, **36:** 646-660.

57. Turner R, Peden JJr, O'Brien K: **Patient-carried card prompts vs computer-generated prompts to remind private practice physicians to perform health maintenance measures.** *Archives of Internal Medicine* 1994, **154:** 1957-1960.

58. Urban N, Taplin S, Taylor V, Peacock S, Anderson G, Conrad D *et al*.: **Community organization to promote breast cancer screening among women ages 50-75.** *Preventive Medicine* 1995, **24:** 477-484.

59. Van der Weijden T, Grol R, Knottnerus J: **Feasibility of a national cholesterol guideline in daily practice. A randomized controlled trial in 20 general practices.** *International Journal for Quality in Health Care* 1999, **11:** 131-137.

60. van Essen G, Kuyvenhoven M, de Melker R: **Implementing the Dutch College of General Practitioner's guidelines for influenza vaccination: an intervention study.** *British Journal of General Practice* 1997, **47:** 25-29.

61. Vinicor F, Cohen S, Mazzuca S, Moorman N, Wheeler M, Kuebler T *et al*.: **DIABEDS: a randomized trial of the effects of physician and/or patient education on diabetes patient outcomes.** *Journal of Chronic Diseases* 1987, **40:** 345-356.

62. Vissers M, Hasman A, van der Linden C: **Protocol processing system (ProtoVIEW) to support residents at the emergency ward.** *Computer Methods & Programs in Biomedicine* 1995, **48:** 53-58.

63. Watson M: *The development, implementation and evaluation of prescribing guidelines in general practice.* Primary Health Care and Epidemiology,University of Bristol; 1998. PhD.
