# Supplementary material for: A systematic review of the use of theory in the design of guideline dissemination and implementation strategies and interpretation of the results of rigorous evaluations
Source: Implement Sci. 2010 Feb 9;5:14. doi: 10.1186/1748-5908-5-14 (PMC2832624; doi:10.1186/1748-5908-5-14)
Supplement: Additional file 2 — Glossary of theories/frameworks used. Brief descriptions of the identified theories and frameworks. [file 1748-5908-5-14-S2.DOC]

## Additional file 2 Glossary of theories/frameworks used

(Authors’ note: the referees requested further information about the theories identified within the systematic review. Additional file 2 is a summary of an Appendix from the PhD thesis of PD. It briefly summarises the concepts of key theories and provides references to key texts for readers interested in learning more about the thesis).

Each of the theories used within the review is described within this section and are listed in alphabetical order. In some instances it was necessary to assign descriptive labels to the instances of theory use if they were not explicitly named by the authors of the papers.

The descriptions of theories are based on the information contained in the papers themselves. Every attempt was made to chase up references contained within the papers to provide a fuller description of the theories used. Where this has not been possible (either because no reference was given or the reference supplied could not be retrieved), searches were made in the reference sections of general psychology texts, as well as social science or psychology search engines.

The amount of information provided for each individual theory is a reflection of the amount of information available and the degree of use within the review.

## Academic detailing

Academic detailing is a method of educational outreach based on principles of ‘detailing’ – the promotional activities used by pharmaceutical companies’ sales representatives to influence prescribing[1]. Such visits generally lasts less than ten minutes and contain several effective strategies for behaviour change that serve to reinforce and complement each other. The techniques involved in academic detailing are based on research in the fields of marketing, adult learning, diffusion of innovations, persuasive communication and behaviour modification techniques.

According to marketing research, success is viewed as likely only when the needs, perceptions and requirements of the target group are identified and met. Drawing on this area of research, Soumerai and Avorn underline the importance of understanding the physicians’ motivations, considering factors such as attitudes towards particular diseases or patients, habits, peer influence, patient demand, time etc.[1]

The involvement of ‘opinion leaders’ (those individuals who tend to be early adopters of innovations and are important and respected sources of influence for others) is based on diffusion of innovation theory [2] (a fuller description of this theory can be found below). The involvement of opinion leaders in the design and implementation of interventions can result in rapid communication of ideas, and reference to these individuals within the detailing visit can also increase its effectiveness.

Persuasion research stresses the importance of two-way communication and presenting both sides of an issue to increase physician involvement and to tailor the message to the needs and values of the physician. The authors also observe that research and practice in the field of adult learning supports the role of learner involvement in attaining educational and behavioural objectives as well as achieving greater learner satisfaction.

Finally, academic detailing also draws on the behaviour modification techniques of repetition and reinforcement to further emphasise the key ideas that the detailer wishes to convey to the physician. Repetition of a few major points is a technique that aids memory retention and promotes behaviour change. These can also be supported by educational materials which emphasise the main ideas in a straightforward way. Feedback of improved behaviour can be used as a positive reinforcer to increase the persistence of behaviour change.

Based on the techniques outlined above, the most important techniques of academic detailing include:

1. Conducting interviews to investigate baseline knowledge and motivations
2. Focusing programs on specific categories of physicians as well as on their opinion leaders
3. Defining clear educational and behavioural objectives
4. Establishing credibility through a respected organizational identity, reinforcing authoritative and unbiased sources of information, and presenting both sides of controversial issues
5. Stimulating active physician participation in educational interactions
6. Using concise graphic educational materials
7. Highlighting and repeating the essential messages
8. Providing positive reinforcement of improved practices.

## Behaviour modification techniques

Behavioural approaches (e.g. [3]) focus on the effects that external stimuli have in shaping behaviour. From this perspective, in order for a behaviour to occur it must be stimulated or cued by a stimulus, and in order for the behaviour to reoccur it should be followed by a rewarding outcome.

Behaviour modification techniques include providing cues to initiate the desired behaviour, and the provision of feedback or rewards to reinforce behaviour in order to strengthen the likelihood of the behaviour being carried out again in the future.

## Continuous Quality Improvement

Continuous Quality Improvement (CQI) has its roots in the industrial-quality management process and involves the application of principles that recognize individuals as components of a system [4]. These systems (as opposed to the individuals that comprise them) are viewed as the unit of analysis in identifying barriers and developing solutions to implement research findings. Hence although CQI interventions frequently have the aim of changing individual practice patterns, they do not directly focus directly on individuals.

CQI uses group decision-making methods. A multi-disciplinary group is convened to employ a structured problem-solving approach to develop improvement strategies. The group then monitors whether its measures of process changes are successful in improving outcomes.

The stages involved in a typical model of CQI include:

1. Define the mission
2. Identify ‘customers’
3. Describe the work process
4. Measure processes
5. Target improvement (i.e. define the problem and identify goals)
6. Diagnose the problem (i.e. identify its root causes)
7. Identify and design improvement
8. Implement improvement
9. Monitor the performance
10. Repeat the cycle of design and implementation of improvement.

## Cybernetic control theory

According to cybernetic theory [5], the essential means of achieving control over performance is through the use of feedback. Through this feedback a system can regulate itself by making a comparison of actual and desired performance, and a subsequent adjustment of operations if a discrepancy is found. Discrepancies can be caused by events outside of the system as well as within the system.

## Diffusion of innovation theory

Diffusion of innovation theory focuses on the way in which new ideas or technologies (innovations) spread through groups or communities.[2] Change is characterised as being a dynamic process that occurs over time.

Originally based on agricultural research to increase production through the introduction of a higher yield seed, the theory has been applied to the uptake of new ideas within medical communities by several researchers (e.g.[6-8]).

Four stages of adoption are identified: the knowledge stage, which involves learning about the innovation; the persuasion stage, in which the individual forms positive or negative attitudes about the innovation; the decision stage, in which the individual tests the acceptability of the innovation; and the final stage, characterised by the adoption or rejection of the innovation. The process is not exclusively unidirectional – an individual can move back and forth between stages, and different people move at different speeds.

Individuals can be classified as innovators, early adopters, early majority, late majority and laggards according to how quickly they adopt new technology and change their behaviour. Diffusion of innovation theory places important emphasis on innovators as “change agents” who identify with the concerns of the community and influence decisions about the adoption of an innovation.

Adoption of the innovation through a community is at first gradual, then eventually the rate of diffusion escalates rapidly in an S-shaped function (when the cumulative number of adopters is plotted against time) until the innovation has been adopted to saturation point.

As with stages of change theories, a range of techniques will be required to encourage different types of individuals to change their behaviour. Similarly, different types individuals may also perceive different barriers and have differing resources.

The theory also highlights characteristics of the innovation that influence the diffusion process: its relative advantage (whether the benefits outweigh the costs), its compatibility (with personal and local norms), its complexity (whether it is simple to use), its trialability, (the extent to which it can be adopted temporarily and abandoned if found to be inadequate) and its observability (how easily one can see whether the expected results are being achieved).

**Elaboration likelihood model**

The Elaboration likelihood model (ELM) is a theory about the processes underlying changes in evaluative judgements about objects, issues and people [9]. According to the model, there are two routes through which persuasion can be achieved to bring about these changes in judgement (commonly conceived of as a change in attitude). The two routes – “central” and “peripheral” refer to attitude changes that are based on different degrees of elaboration i.e. effortful thought on an issue.

The degree to which receivers engage in effortful thinking actually forms a continuum, from cases of extremely high elaboration to those of little or no elaboration. Points along the elaboration continuum are determined by how motivated and able individuals are to assess and elaborate upon the value of the referent issue. Persuasion can take place at any point along the continuum, but the nature of the persuasive process differs as the degree of elaboration varies. To highlight the differences in persuasion processes the ELM makes the broad distinction between the two different routes to persuasion.

The “central route” to persuasion represents the persuasion processes involved when elaboration likelihood is high. Persuasion is achieved through effortful thinking on issue-relevant considerations. By contrast, the “peripheral route” to persuasion represents those persuasion processes involved when elaboration likelihood is low. Such low-effort attitude changes are usually based on simple decision rules or cues (e.g. credibility of the communicator) rather than engaging in issue relevant thinking.

According to the ELM, if persuasive messages are deeply processed through the central route, the resulting attitudes changes are more persistent and more resistant to counter-persuasion than those produced through the peripheral route.

## Field Theory

Lewin was an important figure in psychology who promoted the idea that all human behaviour, including psychological processes, could be studied experimentally in a period dominated by the behaviourist paradigm. According to Lewin’s (1951) field theory, behaviour is a function of personal characteristics and the social situation [10]. This proposition that human behaviour is the function of both the person and the environment could, according to Lewin, be expressed in the symbolic terms:

B= f (P,E)According to its author, Field theory “is probably best characterised as a method: namely a method of analysing causal relations and of building scientific constructs” [10].

Lewin carried out considerable research on group dynamics, based on his belief that the structural properties of the group as a whole are characterized by the relations between parts rather than simply by the properties of the parts themselves.

Lewin demonstrated the effectiveness of using group decision processes in order to change the behaviour of individuals. A group decision helps to remove resistance to change by resolving discrepancies between the standards and expectations of the individual and the group, permits the sharing of information about barriers to the proposed change and techniques to overcome these, and leads to a greater sense of commitment to the change process than if the decision had been imposed from outside of the group.

Force field analysis is a technique developed by Lewin for considering the forces for and against a decision.[10] This technique involves listing all of the forces for or against a proposed change and assigning a score to each. The forces are usually represented in a diagram and can be used to decide whether or not a proposed change is viable. The results of the force field analysis can be used to guide the change process by working to reduce the strength of the forces operating against change, and/or strengthening the forces that are pushing for the change.

## Grol’s 4 step model

This framework for implementing guidelines is based on a four-step process outlined by Grol [11]. The model is based on ideas about how to change behaviour from several disciplines, and includes Diffusion of Innovation theory [2], the Theory of Reasoned Action [12], and Social Cognitive Theory [13]. The paper itself contains very little detail about the theories themselves or how they relate to the implementation process. A fuller description of each of the theories can be found elsewhere in this section. The four steps of the implementation process outlined by Grol can be summarized as follows [11]:

- Orientation
  - Attention and becoming informed about the existence of new guidelines
  - Feeling interest and commitment
- Insight
  - Understanding the guidelines
  - Awareness of (gaps in) in own performance, persuasion of the need to change
- Acceptance
  - Positive attitude to the new guidelines
  - Intention to change, confidence in success
- Change
  - Actual implementation in practice, experimentation
  - Recognition of positive outcomes, maintenance.

Each specific step in the process may be accompanied by specific problems or barriers, which may relate to either the characteristics of the physician or to those of the practice setting. It is important to give consideration to these and adapt interventions to overcome them.

## Goals, Emotions, and Personal Capability Beliefs Theory of Motivation

Ford’s theory of motivation is based around anticipating an individual’s goals in life [14]. According to the theory, people can be motivated to do something if they feel capable of if (personal capability) they can be excited enough (emotions) and it will help them achieve a personal goal (goals).

## Health Belief Model

The Health Belief model was originally developed as a model upon which to base health education programs [15]. The model focuses on the beliefs that predict the likelihood that an individual will carry out a particular health behaviour.

The key health beliefs identified in the model are *perceived susceptibility* (an individuals beliefs about the likelihood of experiencing a particular health problem); *perceived severity* (beliefs about the severity of the consequences of the health problem) and the perceived costs and benefits of carrying out the appropriate health behaviour.

Generally all components of the HBM are treated as independent predictors of behaviour, however susceptibility and severity are often combined conceptually to produce perceived threat, as are costs and benefits of the health behaviour in order to determine evaluation of the effectiveness of the health behaviour to counteract this threat (response effectiveness). Consequently, individuals are likely to carry out a health behaviour if they believe themselves to be susceptible to a particular condition which they perceive to be serious, and if they believe that the benefits of the action to be taken outweigh the costs.

Two additional variables commonly included in the model are cues to action and health motivation. Cues to action are commonly divided into factors that are internal to the individual (e.g. pain) and those which are external (e.g. advice from the doctor). Health motivation refers to an individual’s readiness to be concerned with health matters.

Other influences on behaviour such as demographic variables and other psychological factors (e.g. personality, self-efficacy) are held to exert their effects through changes in the six components specified by the model.

## Information theory

This theory is concerned with the ways in which the human mind processed information, and can be attributed largely to the work of McDonald and colleagues at the Regenstreif Institute where it has been applied to the medical profession particularly in relation to the use of computerised reminders.[16] The theory also draws upon the work of human error theorists.

According to McDonald’s theory, there are limits to man’s capabilities as an information processor and these limits are the cause of random error within activities. When keeping watch for random or infrequent events the human mind inevitably fails to spot all occurrences. Sensory overload, such as that faced by physicians in busy practice settings, heightens the opportunities for such errors. Consequently, many physician errors can be seen as being due to the limits of the capacity of the human mind to process information rather than as indicative of a knowledge deficit.

According to information theory, to eliminate such errors one must commit more time to the processing of relevant data. Many of the physicians’ informational tasks are rote and repetitive and could, according to McDonald, be performed by computers given the necessary decision logic. In this way the computer relieves the physician of the processing time necessary to attend to the relevant data, leaving them free to carry out other tasks.

**Knowledge- attitudes-Practice (KAP)**

The KAP model is not specifically associated with the work of one individual, but is a commonly held view of how to change practice. According to this model practice can be changed by increasing knowledge. The increase is knowledge is proposed to change attitudes so that an individual is more predisposed to perform the behaviour.

## Learning styles

Learning styles reflect individual differences in the ways in which people prefer to learn new information. An individual’s preferred learning style is the product of factors such as personality, prior experiences, and personal circumstances. Two instruments to determine individual learning styles will be briefly described here.

Honey and Mumford’s Learning Styles questionnaire is commonly used in adult education and examines educational preferences in terms of context and content.[17] Four types of learning style can be determined: Activists (like novel experiences, and are open-minded); Theorists (like logical complexity); Reflectors (like to think things through); and Pragmatists (like to try things out). Every learner is a thought to be a mixture of these extremes.

Guglielmino’s Self-Directed Readiness to Learn Scale is a measure of how motivated individuals are to learn (1977, Doctoral dissertation, University of Georgia; cited in Onion [18]). The scale provides a score representing an individual’s readiness to learn, assessed from the perspective of the learner himself or herself.

**Organizational development (The “Criteria participation model”)**

According to organizational development theorists (e.g. Kahn [19]) the most effective way to bring about organizational change is by using methods which alter people’s knowledge and attitudes about each other and their responsibilities. Individual behaviour change becomes possible as employees participate in developing group performance norms, and these in turn become performance regulators.

The “Criteria participation model” is based on the principles outlined above and involves audit and feedback together with physician participation in setting the audit criteria. Within the model, audit is viewed as a method of highlighting individual physician’s knowledge or skills deficits. Participation in setting audit criteria is proposed to assist physicians in updating any knowledge deficits and the act of agreeing audit criteria in a group setting should promote feelings of peer pressure to conform to the standards set.

**Patient care appraisal model**

The patient care appraisal model is a model of educational intervention developed by Davidson, Lein and Kelday [20] based on the earlier work of Schwarz [21]. It comprises the following five steps:

1. Selecting medical conditions for study
2. Generating criteria of care for those conditions
3. Examining physicians’ records for conformity with those criteria
4. Reporting the results of the audits to the physicians involved
5. Repeating the audit later to assess behavioural change

The model was developed following criticisms of studies of physician continuing medical education, in which a common observation was that the increase in knowledge found post-intervention contrasted with no improvement in patient care. Goldfinger attributes these findings to the focus on studying the effects of CME as “modular units” without considering other sources of information and influence such as physician motivation and the professional environment [22]. The crucial aspect of this model that builds upon the traditional CME models is the full involvement of participants in planning remedial learning activities.

## PRECEDE

The PRECEDE model is not a theory as such, rather it is a conceptual framework or planning model that can be used to guide the choice and implementation of appropriate intervention strategies [23]. Originally developed to enhance the quality of health education interventions, the PRECEDE acronym stands for Predisposing, Reinforcing, and Enabling Constructs in Educational Diagnosis and Evaluation.

A fundamental idea within the PRECEDE model is the principle of participation. Every effort should be made to include the target audience at every stage of the planning, implementation and evaluation of the intervention program.

The PRECEDE model is a five-phase process which acknowledges that health behaviours are complex and influenced by a variety of individual and environmental factors. The model begins at the end point (Phase 5), focusing on the outcome of interest and then working backwards in order to identify the best steps towards achieving that outcome. Phases 1 –3 are concerned with identifying the social, epidemiological, behavioural and environmental factors which influence the target population in relation to the chosen outcomes studied. Phase 4 (educational and organisational diagnosis) will be described in more detail in this section as this is the phase which the studies reviewed have focused on.

Phase 4, the educational and organisational diagnosis, identifies those factors that must be changed in order to initiate and sustain the process of change. These factors can be seen as the immediate targets or objectives of the change program, which must be modified if the necessary changes are to occur. The PRECEDE model identifies three categories of factors which influence behaviour. Any given behaviour can be explained as a function of the combined influence of these three types of factors.

*Predisposing factors* are the antecedents to a behaviour that provide the rationale or motivation for that behaviour e.g. knowledge, attitudes, existing skills, perceived needs and self-efficacy beliefs. Personality characteristics and demographic factors could also be conceived of as predisposing factors, however these are given little attention within this phase of the model as these factors are not easily modified. *Enabling factors* are antecendents that enable the motivation to act to be realised. These include the resources and new skills necessary to carry out the required behaviour and any organizational actions necessary to make the environment more facilitating. *Reinforcing factors* are those that appear following the behaviour and provide continuing incentives for the behaviour to become permanent.

Relationships exist between the three factors. A person has an initial motivation (predisposing factor) to carryout a behaviour, however they cannot do so unless they possess the resources or skills (enabling factors) to do so. The performance of the behaviour will be followed by a reaction, which can be emotional, physical or social (reinforcing factor). This reinforcement may serve to strengthen behaviour, resources (enabling factors) and motivation (predisposing factors). The presence of enabling factors in the environment provides cues and heightens awareness and other factors predisposing the behaviour.

The purpose of the educational and organizational diagnosis phase is to select the factors which, if modified, will bring about the desired behaviour change. Three steps are involved in this process: identifying and sorting factors into the three categories; establishing priorities between categories; and establishing priorities within categories. These priorities should be established according to the relative importance and changeability of the factors identified. Although the PRECEDE model is not a theory per se, it can be used to guide the choice and application of appropriate behaviour change theories

An extension of the PRECEDE model has been the addition of the PROCEED component in 1991 (Policy, Regulatory, and Organizational Constructs in Educational and Environmental Development) which recognizes the need to consider the implementation and evaluation of the intervention. Within this, the predisposing, enabling and reinforcing components identified in phase 4 can be measured as part of the process and impact evaluation to assess the extent to which the intervention has changed these factors.

Davis et al carried out a systematic review of the impact of continuing medical education (CME) interventions on physician performance and health care outcomes [24]. An intervention was considered educational if it “primarily consisted of the transfer of information to targeted physicians and was intended to affect physician performance through persuasion”.

Based on the predisposing, enabling and reinforcing factors described in phase 4 of the PRECEDE model, Davis et al classified CME interventions included in their review as being predisposing (communicating or disseminating information), enabling (facilitating the desired change in the practice site) and reinforcing (by reminders or feedback). From these four intervention types were derived:

1. Those using predisposing factors only
2. Predisposing plus enabling factors
3. Predisposing plus reinforcing factors
4. All three categories or multifaceted intervention

**Table. 1. Intervention types classified using Precede**

| Intervention type | CME interventions |
| --- | --- |
| Predisposing | Academic detail visits; computer-generated information; consultations; didactic presentations; lectures; knowledge testing; needs assessment; printed materials; traineeship workshops; tutorials; small groups |
| Predisposing and Enabling | Clinical policy and practice guidelines; patient education materials or instruction; information from patients; practice protocols; algorithms; flow charts |
| Predisposing and Reinforcing | Feedback; Reminders |
| Multifaceted interventions | Chart reviews and chart-stimulate recall; influential educational leaders; clinical opinion leaders |

Interventions using only predisposing elements were less likely to change physician performance and had little or no effect on patient outcomes (a finding which supports previous criticisms of these types of CME strategies, and the recommendations of the PRECEDE authors [23]). In contrast, those studies that included enabling and/or reinforcing strategies were more effective in changing outcomes

## Rule-based expert system approach

Expert systems are computerized systems which can be used to support physicians processing of information and facilitate decision making based on these information processing procedures.

According to Buchanon expert systems should be able to provide a solution to a problem at the same level of performance as a human expert; employ symbolic and heuristic reasoning rather than numeric and algorithmic procedures; store knowledge separately from inference procedures; and provide explanations of their reasoning [25].

Two main types of expert system can be distinguished: rule-base systems, where operations are performed subject to a set of rules obtained from specialists in the field; and probabilistic systems, where evidence is weighed and the best course of action is calculated according to Bayes Theorem.

Although rule-based systems are simpler and cheaper than probabilistic systems they reflect the biases and logical errors of human thinking (Baron 1988) and do not allow the separation of knowledge and inference. Much of the physician consultation process is not suited to knowledge representation in a rule-based sense. However, a review of the use of decision support software amongst GPs identified three areas in which a rule-based system can be used for decision support during consultations: covering gaps in knowledge; fail safes, such as drug interaction warnings; and tools for education, reference or communication [26].

## Social Cognitive Theory

Social cognitive theory was developed by Bandura and suggests that behaviour is determined primarily by incentives and expectancy beliefs.[13,27] Incentives (i.e. reinforcement) refer to the consequences of performing the behaviour. An individual is more likely to perform a behaviour that results in desirable consequences. Three types of expectancy can be identified.

- Situation-outcome expectancies refer to an individual’s beliefs about the extent to which outcomes are cued by environmental events and are therefore outside of personal control.
- Action-outcome expectancies are concerned with the belief that outcomes follow from personal action
- Perceived self-efficacy refers to an individual’s beliefs about their capabilities to perform a specific action required bringing about the desired outcome.

According to SCT therefore, an individual is likely to perform a behaviour if they believe that a rewarding outcome will result, if they believe that they have control over that outcome, perceive few external barriers, and have confidence in their own capabilities to perform the behaviour.

The self-efficacy component is usually the strongest predictor of behaviour and is often the main focus of research efforts. Those individuals with strong self-efficacy beliefs are said to develop stronger intentions to perform the behaviour, work harder towards achieving their goal, and persist longer when faced with barriers and obstacles.

According to Bandura self-efficacy can be conceptualised and measured along three dimensions.[27] Strength refers to an individual’s belief that they can perform a specific behaviour. Magnitude refers to individuals’ expectations about their level of performance depending on the degree of difficulty of the behaviour (those with low expectations will only believe themselves capable of performing relatively simple behaviours). Finally, generality refers to the generality of self-efficacy beliefs across situations or behaviours. Most research on self-efficacy tends to focus on the measurement of the strength of the self-efficacy belief.

## Shotgun method

The shotgun method describes a combination of interventions aimed at stimulating internal and external motivations of physicians [28]. Internal motivation can be stimulated by competence-orientated strategies (the provision of educational materials, and group education methods) and performance-orientated strategies (feedback of performance). External motivation can be promoted by peer review and practical support.

## Social influence Theory

The social influence perspective on practitioner behaviour change developed by Mittman, Tonesk and Jacobson is based on theory and research in social psychology, sociology, health behaviour change and health services research [29]. The term social influence refers to “the process in which the behaviour of one person has the effect or intention of changing how another person behaves, feels, or thinks about something” [29].

Mittman et al argue against traditional models of physician behaviour in which decisions and actions are based on cost/benefit analyses of competing actions in relation to attaining specific goals [29]. In contrast, the authors argue that practitioner decisions are “guided by habit and custom; by assumptions, beliefs, and values held by peers; and by prevailing practices and social norms that define appropriate behaviour” [29].

Several behaviour change/guideline implementation strategies are described from a social influence perspective, including academic detailing, consultation and peer discussion, Continuous Quality improvement, participation in guidelines development and the use of opinion leaders. Mittman et al describe these strategies in terms of the primary mechanism through which behaviour change occurs (by modifying group norms or individual beliefs); whether the strategy primarily involves the transfer of knowledge and information or the transfer of norms and values; and the level of effort required in terms of time, costs and other resources [29].

## Social Learning Theory

Social Learning theory has arisen from the work of several researchers, most notably Bandura [30]. Whilst earlier models of behaviour focused on the influence on environmental factors in shaping behaviour, social learning theory posits a more complex, reciprocal relationship between the individual and his or her environment. Bandura refers to this relationship as *reciprocal determinism* and emphasises the way in which our behaviour and our environment continually interact and influence each other [30].

A key element of social learning theory is *observational learning*. Earlier models of behaviour describe the way in which behaviour is shaped by its consequences, with positive consequences increasing the likelihood of the behaviour reoccurring and negative consequences decreasing this likelihood. Observational learning extends this idea to learning through observation of the behaviour of others and the resulting consequences of their behaviour. Through this method of learning individuals build up expectancies about the likely consequences of performing particular patterns of behaviour themselves.

Through the principles of reciprocal determinism and observational learning, social learning theory emphasises the importance of the social environment in shaping behaviour.

Bandura further expanded upon his ideas about the expectancy beliefs individuals develop with regard to different patterns of behaviour (incorporating beliefs about their own abilities to carry out behaviours) to develop Social Cognitive theory, which is also described in this section.

## Stages of change theories

Stages of change theories are based on the idea that behaviour change is a dynamic process as opposed to an event. The most popular stages of change model is the Transtheoretical model (TTM) proposed by Prochaska and DiClemente and often referred to as the “readiness to change” model [31]. Originally developed in relation to smoking cessation behaviour, the model identifies 5 different stages of change through which individuals can pass in changing behaviour.

Individuals at the *precontemplation* stage are those who are not even considering changing their behaviour, or who consciously intend not to change. In the *contemplation* stage individuals begin to consider making a change in their behaviour. At the *preparation* stage the individual has made a serious commitment to change their behaviour. The behaviour change is initiated in the *action* stage, and in the fifth, *maintenance,* stage the behaviour change is sustained. (A sixth stage of *termination* has been identified for some behaviours e.g. overcoming addiction).

An individual can enter the change process at any stage. Some individuals move through the stages faster than others and some appear to get stuck at a particular stage. Sometimes individuals may slip back to a prior stage.

A key aspect of the model is that different intervention strategies are required to help individuals at different stages of change. For example, in the precontemplation and contemplation stages individuals may benefit from the provision of information, whereas in the preparation and action stages individuals may require different help such as skills training and advice on how to overcome barriers. Those at the maintenance stage can be helped by environments that provide support and rewards to reinforce their behaviour.

## Theory of Reasoned Action

According to the Theory of Reasoned Action the proximal determinant of behaviour is an individual’s *intention* to carry out the behaviour in question [12]. Intentions can be conceived of as a person’s motivation or determination to apply effort towards performing that behaviour. Behavioural intentions are themselves determined by *attitude* (the individual’s overall evaluations of the behaviour in question) and *subjective norms* (the extent to which the individual believes that significant others think that they should perform the behaviour) [12].

The TRA is proposed to explain volitional behaviours (i.e. those which are the individual’s control). The theory of planned behaviour adds an additional component of perceived behavioural control in order to extend the theory to non-volitional behaviours as well.

## Treatment theory

Lipsey’s treatment theory is one of a variety of evaluative methods which can be described as a theory-driven evaluation.[32] In this type of evaluation the analysis is directed by a theory of how the participants involved and the various components of the intervention all interact to produce the change in outcome. This theory can then be used to guide data collection to determine whether the program is in fact operating in the ways proposed. This type of investigation is often seen as analogous to opening the “black box” of the intervention.

Lipsey proposes that what goes on within the black box can be investigated using a small theory of treatment (small in that such a theory attempts to explain specific intervention processes as opposed to a large theory of general phenomena) to describe the processes through which an intervention, or treatment, is expected to have effects on a specified target population [32]. Elements that need to be defined within a treatment theory include [32]:

1. A definition of the problem specifying its aetiology if possible, its magnitude, its consequences, and the populations and circumstances to which it applies.
2. Specification of what are believed to be the critical inputs, the interrelationships among these inputs, a conception of the strength of treatment, and specification of the minimal operationlisation necessary in order to deliver the treatment at an effective strength.
3. The mechanisms by which the planned treatment in supposed to have its effects, including the intervening or mediating variables on which the process is contingent, and the crucial interactions with individual differences, timing, mode of delivery, or other relevant circumstances.
4. Specification of the desired output, including the minimal magnitude of effect necessary, the maximal magnitude thought likely, and the timing with which such effects are expected to occur.

The development of a treatment theory can come from sources such as existing theory from relevant disciplines, from earlier pilot studies carried out before the evaluation of the program, and can be developed from the implicit theories of program personnel, relevant clinical practitioners, or intended recipients of the program.

## The vividness criterion

Vividly presented information is generally thought to be more persuasive and to have more impact on judgements than nonvividly presented information. According to Tversky and Kahneman, under situations of judgemental uncertainty individuals use shortcuts, or heuristics, to make inferences [33]. According to the “availability” heuristic, individuals judge the frequency of an event by how readily it is brought to mind. An availability bias is said to exist when the ease with which information is brought to mind is influenced by irrelevant factors such as recency, or memory biases.

Nisbett and Ross (1980) discuss what they refer to as the “vividness criterion” as being one source of availability bias [34]. They argue that vividly presented material is more effectively processed at the information encoding stage and is therefore more likely to be available to memory than nonvivid information when judgements are made.

Information can be described as vivid to the extent that it is “a) emotionally interesting; b) concrete and imagery-provoking; and c) proximate in a sensory, temporal, or spatial way” [34]. Direct experience is regarded as more vivid than nondirect experience, and case histories are thought to be more vivid than other forms of presentation, such as statistical information, because they contain more concrete information and more image-enhancing properties [35].

References for Additional File 3

1. Soumerai SB, Avorn J: **Principles of educational outreach ('academic detailing') to improve clinical decision making.** *JAMA* 1990, **263:** 549-556.

2. Rogers E: *Diffusion of Innovations*. New York: Free Press of Glencoe; 1962.

3. Mazzuca s: **The role of the clinical environment in the translation of research into practice.** *The Diabetes Educator* 1986, **12:** 219-224.

4. Kritchevsky SB, Simmons BP: **Continuous quality improvement. Concepts and applications for physician care.** *JAMA* 1991, **266:** 1817-1823.

5. Beer S: *Decision and control*. London: Whiley; 1966.

6. Coleman J, Katz E, Rogers E: *Medical Innovation: A diffusion study*. New York: Bobbs-Merrill; 1966.

7. Greer A: **The state of the art versus the state of the science. The diffusion of new medical technologies into practice.** *International Journal of Technology Assessment in Health Care* 1988, **4:** 5-26.

8. Haines A, Jones R, .: **Implementing findings of research.** *British Medical Journal* 1994, **308:** 1488-1492.

9. Petty R, Cacioppo J: *Communication and persuasion: central and peripheral routes to attitude change*. New York: Springer-Verlag; 1986.

10. Lewin K: *Field Theory in social Science*. New York: Harper and Row; 1951.

11. Grol R: **Implementing guidelines in general practice care.** *Quality in Health Care* 1992, **1:** 184-191.

12. Fishbein M, Ajzen I: *Belief, attitude, intention and behaviour: an introduction to theory and research*. Reading, Massachusettes: Addison-Wesley; 1975.

13. Bandura A: *Social foundations of thought and action: a social cognitive theory*. Englewood Cliffs, NJ: Prentice Hill; 1986.

14. Ford M: *Motivating humans. Goals, emotions and personal agency beliefs*. Newbury Park, CA: Sage; 1992.

15. Janz N, Becker M: **The health belief model: A decade later.** *Health Education Quarterly* 1984, **11:** 1-47.

16. McDonald CJ: **Protocol-based computer reminders, the quality of care and the non-perfectability of man.** *N Engl J Med* 1976, **295:** 1351-1355.

17. Honey P, Mumford A: *The manual of learning styles*, Third edn. edn. P. Honey, Maidenhead; 1992.

18. Onion C: *Changes inmedical practice following superficial and deep processing of evidence: a controlled experiment in clinical guideline implementation.* University of Liverpool; 1997. PhD.

19. Kahn R: **Organizational development" Some problems and proposals.** *Journal of Applied Behavioral Sciences* 1974, **10:** 4.

20. Davidson R, Lein J, Kelday J: **Quality assurance in the provision of hospital care. Case study. Audit network aids continuing medical education.** *Hospitals* 1974, **48:** 85-88.

21. Schwarz M: **WAMI: A concept of regionalized medical education.** *Journal of Medical Education* 1973, **56:** 103-110.

22. Goldfinger S: **Continuing medical education: The case for contamination.** *New England Journal of Medicine* 1982, **306:** 540-541.

23. Green LW, Kreuter MW: *Health promotion planning: an educational and environmental approach*, 2nd edn. Mountain View, California: Mayfield Pub Co; 1991.

24. Davis DA, Thomson MA, Oxman AD, Haynes RB: **Evidence for the effectiveness of CME. A review of 50 randomized controlled trials.** *JAMA* 1992, **268:** 1111-1117.

25. Buchanon B, Smith R: **Fundamentals of Expert systems.** *Annals of the Royal Computing Society* 1988, **3:** 23-58.

26. Timpka T: **Introducing hypertext in primary care: a study on the feasibility of decision support for practitioners.** *Computing methods in biomedicine* 1989, **29:** 1-13.

27. Bandura A: **Self-efficacy: toward a unifying theory of behavioral change.** *Psychol Rev* 1977, **84:** 191-215.

28. Lomas J, Haynes RB: **A taxonomy and critical review of tested strategies for the application of clinical practice recommendations: from "official" to "individual" clinical policy.** *Am J Prev Med* 1988, **4:** 77-94.

29. Mittman B, Tonesk X, Jacobson P: **Implementing clinical practice guidelines: social influence strategies and practioner change.** *Quality Review Bulletin* 1992, **18:** 413-422.

30. Bandura A: *Social Learning Theory*. Englewood Cliffs, NJ: Prentice Hall; 1977.

31. Prochaska J, Diclemente C: *The transtheoretical approach: crossing traditional boundaries of change*. Homewood, IL: J. Irwin; 1984.

32. Lipsey MW: **Theory as method: Small theories of treatments.** *New directions for program evaluation* 1993, **57:** 5-38.

33. Tversky A, Kahneman D: **Judgement under uncertainty: heuristics and biases.** *Science* 1974, **185:** 1124-1131.

34. Nisbett R, Ross L: *Human interface:strategies and shortcomings of social judgement*. Englewood Cliffs, NJ: Prentice-Hall; 1980.

35. Taylor S, Thompson S: **Stalking the elusive "vividness" effect.** *Psychological Review* 1982, **89:** 155-181.
